# Dual-functional titanium implants via polydopamine-mediated lithium and copper co-incorporation: synergistic enhancement of osseointegration and antibacterial efficacy

**DOI:** 10.3389/fbioe.2025.1593545

**Published:** 2025-05-12

**Authors:** Jun Li, Bo Jiang, Liu Yang, Pu Zhang, Jingwen Wu, Yalan Yang, Yan Yang, Guiling Wang, Jie Chen, Ling Zhang, Shiqin Huang, Lingli Zhang, En Zhang

**Affiliations:** NMPA Key Laboratory for Quality Monitoring of Narcotic Drugs and Psychotropic Substances, Chongqing Institute for Food and Drug Control, Chongqing, China

**Keywords:** Ti6Al4V, surface modification, metal ions, antibacterial, osseointegration

## Abstract

**Introduction:**

Orthopedic implant failure due to inadequate osseointegration and infection remains a critical challenge. To address this, we engineered a polydopamine (PDA)-mediated dual-functional platform for lithium (Li^+^) and copper (Cu^2+^) co-incorporation on titanium alloy (Ti6Al4V) implants, aiming to synergize osteogenic and antibacterial properties through a scalable surface modification strategy.

**Methods:**

PDA coatings were polymerized onto polished Ti64 substrates, followed by sequential immersion in LiCl (800 μM) and CuCl_2_ (10 μM) solutions to construct Li^+^/Cu^2+^ co-doped surfaces (PDA@Li 800-Cu 10). In vitro assays assessed MC3T3-E1 pre-osteoblast proliferation (CCK-8), osteogenic differentiation (ALP activity, RT-PCR for *ALP/Axin2*), and antibacterial activity against S. aureus and E. coli (live/dead staining, CFU assays). In vivo efficacy was evaluated in a rat femoral defect model via micro-CT and histology.

**Results and discussion:**

Li^+^-functionalized surfaces (PDA@Li 800) enhanced osteoblast proliferation and osteogenesis via Wnt/β-catenin activation. Cu^2+^-loaded coatings (PDA@Cu 10) eradicated >99% bacteria but moderately suppressed osteogenic markers. The dual-doped PDA@Li 800-Cu 10 surface resolved this bioactivity conflict, maintaining antibacterial efficacy comparable to PDA@Cu 10 while elevating the osteogenic capacity of Cu^2+^-only modified surfaces. In vivo, dual-modified implants eliminated bacterial colonization within 72 h and significantly increased peri-implant bone volume (BV/TV) in comparison to Ti64 controls, outperforming PDA-only counterparts. By harmonizing Li-driven osteoinduction and Cu-mediated bactericidal action through a scalable PDA platform, this work advances a transformative strategy for next-generation orthopedic and dental implants, simultaneously addressing infection risks and bone regeneration demands.

## 1 Introduction

Titanium (Ti) and its alloys, particularly Ti6Al4V, are widely utilized in orthopedic and dental implants due to their superior mechanical properties, corrosion resistance, and biocompatibility (Marin and Lanzutti, 2023; Cui et al., 2024). Despite their clinical success, implant failure rates remain significant, with about 5% of orthopedic and 10% of dental implants requiring revision surgeries due to poor osseointegration or infection in 10 years follow-up (Stauss et al., 2024). The global economic burden of implant-associated infections exceeds $1.85 billion annually in the U.S. alone, underscoring the urgent need for advanced implant development that enhances bone regeneration while mitigating microbial colonization (Li et al., 2020; Premkumar et al., 2021). Recent research has focused on surface modification strategies, for example, bioactive metal ions including copper (Cu), silver (Ag), silicon (Si) and lithium (Li), into implant surfaces to simultaneously address these challenges (Yang et al., 2023; Raju and Biswas, 2025).

Metal ions like Cu and Li offer dual-functional benefits: Cu exhibits potent antibacterial effects by disrupting bacterial membranes and generating reactive oxygen species (ROS), while Li promotes osteogenesis via activation of the Wnt/β-catenin signaling pathway (Qiu et al., 2022; Zhang et al., 2024c; Raju and Biswas, 2025). Zhuang et al. reported that the incorporation of 5%Cu to Ti6Al4V to form Ti6Al4V-Cu, achieved stable and continuous Cu^2+^ release, meanwhile, *in vitro* and *in vivo* studies indicated its 99.47% antibacterial rate against *S. aureus* (*Staphylococcus aureus*) (Zhuang et al., 2021). Similarly, Li-doped coatings through micro-arc oxidation not only increased the osteogenic differentiation of MC3T3-E1, but also medicated nerve-induced bone regeneration, as reported by Zhang Q. et al. (2024). However, conventional methods for ion immobilization, such as plasma spraying, sol-gel deposition, and electrochemical anodization, face limitations in controllability, stability, and biocompatibility (Savignac et al., 2018; Koshuro et al., 2023; Raju and Biswas, 2025). Plasma spraying, for instance, achieves high ion-loading capacity but often compromises uniform surface modification, especially for complex porous scaffolds (Bandyopadhyay et al., 2023). Sol-gel coatings enable tunable ion release but exhibit poor adhesion unless elaborate process optimization (Savignac et al., 2018).

Polydopamine (PDA), a bioinspired polymer derived from mussel adhesive proteins, has emerged as a superior platform for metal ion immobilization due to its universal adhesion, biocompatibility, and redox-active surface (Wu et al., 2022). Unlike physical deposition techniques, PDA operates under mild aqueous conditions, preserving substrate microstructure while enabling precise control over ion loading and release kinetics (Wang et al., 2017; Zhu et al., 2023). The presence of catechol and amine functional groups of dopamine enables robust adhesion across diverse substrates, including noble metal, oxides, polymers, semiconductors and ceramics, while facilitating post-functionalization with molecular building blocks, macromolecular and metal ions (Lee et al., 2007). Recently advancements highlight PDA’s utility in delivering therapeutic ions including Zn^2+^ (Pan et al., 2021; Wang T. et al., 2022), Sr^2+^ (Chung et al., 2024), Ag^+^ (Zhang et al., 2024d), Cu^2+^ (Huang et al., 2024) and Au^2+^ (Lee et al., 2018), to enable immunoregulation, antibacterial and osteoinduction in bone tissue engineering. For instance, Wang et al. demonstrated that Zn^2+^-loaded PDA coating on titanium surfaces significantly reduced *S. aureus* biofilm formation by 95.1% with sustained Zn^2+^ release over 7 days (Wang et al., 2019). Similarly, Yang et al. achieved 90.3% eradication against *S. aureus* and 21 days of sustained Cu^2+^ release via Ag nanoparticle immobilization on PDA-functionalized PEEK surfaces (Yang et al., 2023). Recent studies further underscore the potential of multi-ion combinatorial strategies: Huang et al. co-delivered Cu^2+^ and Ag^+^ on titanium to achieve a dual antibacterial and anticoagulant functionality (Huang et al., 2024), while Yang et al. synergized silver and manganese to promote antibacterial and osseointegration properties of PEEK implants (Yang et al., 2023). These efforts reflect a paradigm shift toward leveraging PDA’s coordination chemistry for multifunctional ion delivery.

Despite these advances, lithium (Li^+^) delivery via PDA-mediated systems remains unexplored, even as combinatorial approaches gain traction. While studies have paired ions such as Mg^2+^/Ce^3+^ (Chen et al., 2025) and Mn^2+^/Ag^+^ (Yang et al., 2023), the synergistic potential of Li^+^—a potent osteogenic and anti-inflammatory agent—with complementary ions like Cu^2+^ remains unaddressed. Building on PDA’s covalent/non-covalent binding mechanisms, we hypothesize that co-immobilizing Li^+^ and Cu^2+^ on titanium implants could concurrently enhance infection resistance, especially at the early-stage post-operation and promote osteogenesis over the healing period mediated by the sustained released bioactive Li^+^ and Cu^2+^. Current single-ion systems, such as Cu^2+^-PDA coatings (Wang et al., 2017) or Li^+^-doped biomaterials (Peng et al., 2021; Qiu et al., 2022), lack the multifunctionality required for infected bone defect repair. This study pioneers a Li^+^-Cu^2+^ dual-delivery PDA coating on Ti6Al4V, hypothesizing that Li^+^‘s Wnt/β-catenin signaling activation and Cu^2+^‘s broad-spectrum antibacterial properties will synergistically promote osseointegration and mitigate infection. Through systematic *in vitro* evaluation of MC3T3-E1 responses (proliferation, osteogenic differentiation) and antibacterial efficacy against *E. coli* and *S. aureus*, we optimize ion concentrations to balance bioactivity and cytotoxicity. Subsequent *in vivo* validation in a rat femoral defect model with bacterial inoculation assess infection suppression and peri-implant bone regeneration, providing critical insights into dual-functional implant design.

## 2 Materials and methods

### 2.1 Sample preparation

Ti6Al4V (Ti64) disks (14.0 mm diameter, 1.0 mm thickness) for *in vitro* studies and rods (1.5 mm diameter, 10 mm length) for *in vivo* implantation were wire-cut from wrought Ti64. The disks were mechanically polished using 180-grit silicon carbide grinding paper (Struers) to achieve uniform surface topography. Before further treatment, all substrates underwent sequential ultrasonic cleaning in acetone, 95% (v/v) ethanol, and Milli-Q water for 10 min each to eliminate organic and particulate contaminants, followed by air-drying under laminar airflow.

Polydopamine coating and metal ion immobilization: Polydopamine (PDA) coating was deposited by immersing substrates in a Tris-HCl-buffered solution (10 mM, pH 8.5) containing 2 mg mL^-1^ dopamine hydrochloride (Sigma-Aldrich, H8502) under constant magnetic stirring (100 rpm, 24 h, 25°C). After coating, substrates were ultrasonicated in Milli-Q water (5 min) to remove loosely adhered particles and dried under controlled airflow. For copper ion functionalization, PDA-coated samples were immersed in CuCl_2_ solutions (0.1, 0.5, 2, and 10 mM in Milli-Q water; Sigma-Aldrich, 751,944) for 12 h, rinsed thoroughly, and dried to yield PDA@Cu 0.1, PDA@Cu 0.5, PDA@Cu 2, and PDA@Cu 10. Lithium-ion immobilization followed an analogous protocol using LiCl solutions (50, 200, and 800 mM), producing PDA@Li 50, PDA@Li 200, and PDA@Li 800.

### 2.2 Surface characterization

Surface morphology and elemental composition were analyzed using field-emission scanning electron microscopy (FE-SEM, Zeiss Ultra 55) equipped with energy-dispersive X-ray spectroscopy (EDX, Oxford Instruments). Chemical components were characterized via X-ray photoelectron spectroscopy (XPS; Thermo Scientific K-Alpha+) employing a monochromatic Al Kα source (1486.6 eV, 100 W) under ultrahigh vacuum (<10^−8^ mbar). Survey scans (150 eV pass energy) and high-resolution spectra (50 eV) were acquired, with adventitious carbon (C 1s at 284.8 eV) serving as an internal reference for charge correction. The surface wettability of surfaces was determined by measuring the water contact angle using a contact angle goniometer (KINO Scientific Instrument Inc. United States).

### 2.3 Ion release

The PDA@Li 800-Cu 10 samples were immersed in 2 mL phosphate buffer solution (PBS) and incubated at 37°C to evaluate the ion release profile at 0.5, 1, 2, 3 and 4 days. One milliliter of extracts was collected and 1 mL of fresh PBS was added. The Cu and Li-ion concentrations were quantified using an Inductively Coupled Plasma-Optical Emission Spectrometer (iCAP7400, Thermo Fisher Scientific, United States).

### 2.4 Cell proliferation and osteogenic differentiation on Cu^2+^/Li^+^ incorporated surfaces

#### 2.4.1 Cell culture and seeding

MC3T3-E1 pre-osteoblasts (Subclone 14, Wuhan Pricella Biotechnology) were cultured in α-MEM (Corning, 10-022-CV) supplemented with 10% (v/v) fetal bovine serum (Gibco, 10099141C), 100 U mL^−1^ penicillin, 100 μg mL^−1^ streptomycin, and 0.25 μg mL^−1^ amphotericin B (Beyotime, C0224) at 37°C under 5% CO_2_.

Cells were seeded onto substrates (1 × 10^4^ cells cm^−2^) in 24-well plates (Corning) and switched to osteogenic medium for alkaline phosphatase (ALP) staining, i.e., α-MEM supplemented with 10% FBS (10099141C, Gibco), 0.2 mM ascorbic acid (A8100, Solarbio), 10 mM β-Glycerol phosphate disodium salt pentahydrate (A8100, Solarbio) and 10 nM dexamethasone (D8040, Solarbio) after 24 h. Cells for the remaining evaluation were cultured in an expansion medium and refreshed every 48 h.

#### 2.4.2 Cellular morphology characterization

To assess cellular morphology, immunofluorescence staining of filamentous actin (F-actin) was conducted after 1-day and 7-day culture periods. Cells were fixed in 10% neutral buffered formalin (Sigma-Aldrich) for 10 min at room temperature, followed by permeabilization with 0.1% Triton X-100 (v/v in PBS; Sigma-Aldrich) for 15 min. After three washes with PBS (pH 7.4), samples were incubated with Actin-Tracker Green (C2201S, 1:100 dilution; Beyotime Biotechnology) prepared in 0.1% PBS-Tween 20 (PBST) containing 10% goat serum (C0265; Beyotime Biotechnology) for 1 h at room temperature. Nuclei were counterstained with Hoechst 33258 (B8030, 1 μg mL^−1^; Solarbio) for 10 min under light-protected conditions. Following three additional PBST washes, fluorescence imaging was performed using an Axio Observer A1 inverted microscope (Carl Zeiss Microscopy, Oberkochen, Germany).

#### 2.4.3 Cell proliferation assays

Cell proliferation was quantified using a Cell Counting Kit-8 (CCK-8; C0042, Beyotime, China) assay following the manufacturer’s protocol. Briefly, after 1, 3, and 5 days of culture, the medium was aspirated, and 200 μL of fresh expansion medium containing 10% (v/v) CCK-8 reagent was added to each well. Samples were incubated at 37°C under 5% CO_2_ for 1 h to allow formazan formation. Subsequently, 100 μL of supernatant from each sample was transferred to a 96-well plate, and absorbance was measured at 450 nm using a BioTek Synergy HTX Multimode Reader (Agilent Technologies, Santa Clara, CA, United States). Triplicate measurements were performed for all experimental groups, and data were normalized by subtracting the absorbance of blank controls.

#### 2.4.4 Alkaline phosphatase activity evaluation

Following a 7-day incubation in an expansion medium, cells were lysed using 200 μL of cell lysis buffer (P0013, Beyotime, China) supplemented with 1 mM phenylmethanesulfonyl fluoride (PMSF, ST506, Beyotime, China). ALP activity was quantified using an ALP Assay Kit (P0321S, Beyotime, China) according to the manufacturer’s protocol. Briefly, 50 μL of cell lysate was mixed with 50 μL of substrate solution and incubated at 37°C for 3 h. The reaction was terminated by adding 100 μL of stop solution, and absorbance was measured at 405 nm using a BioTek Synergy HTX Multimode Reader (Agilent, United States). Concurrently, total protein concentration in the lysate was determined via an Enhanced BCA Protein Assay Kit (P0010, Beyotime, China), with absorbance measured at 562 nm. ALP activity was normalized to total protein content and expressed as units per milligram of protein (U mg^−1^).

ALP staining was further performed to determine the ALP activity. After culturing in an osteogenic differentiation medium for 7 days, the samples were fixed with 4% formaldehyde for 10 min and then stained with a BCIP/NBT alkaline phosphatase color development kit (C3206, Beyotime) for 30 min at room temperature.

#### 2.4.5 Gene expression analysis for wnt activation and osteogenic differentiation

The expression of Wnt signaling activation- and osteogenic differentiation-related genes in MC3T3-E1 pre-osteoblasts cultured on distinct sample surfaces was quantified via qRT-PCR following 4-day and 7-day incubation in expansion medium. Total RNA was isolated from cell-laden substrates using TRIzol™ reagent (R0016, Beyotime Biotechnology, China), followed by cDNA synthesis employing a reverse transcription kit (AG11706, Accurate Biology, China). qRT-PCR amplification was performed using a Bio-Rad CFX Connect™ Real-Time PCR Detection System (Bio-Rad Laboratories, USA) with SYBR Green Pro Taq HS Premix (AG11701, Accurate Biology, China), utilizing primer sequences as *GAPDH*-F: TGT​GTC​CGT​CGT​GGA​TCT​GA, *GAPDH*-R: TTG​CTG​TTG​AAG​TCG​CAG​GAG, *Axin2*-F: TGA​GCG​GCA​GAG​CAA​GTC​CAA, Axin2-R: GGC​AGA​CTC​CAA​TGG​GTA​GCT, *ALP*-R: CAC​GGC​GTC​CAT​GAG​CAG​AAC, *ALP*-F: CAG​GCA​CAG​TGG​TCA​AGG​TTG​G. Relative mRNA expression levels of target genes were normalized to the housekeeping gene *GAPDH* and calculated using the 2^−ΔΔCT^ method.

### 2.5 Anti-bacterial efficiency of Cu^2+^/Li^+^ incorporated surfaces

#### 2.5.1 Bacterial culture and inoculation

*Staphylococcus aureus* (strain BMC1-07; Hangzhou Biaomai Biotechnology Co., Ltd., China) and *E*. *coli* (strain BMC3-07; Hangzhou Biaomai Biotechnology Co., Ltd., China) were selected as model Gram-positive aerobic and Gram-negative anaerobic pathogens, respectively. To evaluate the antibacterial performance of functionalized surfaces. The antibacterial efficacy of the material surfaces was quantitatively assessed *in vitro* following the ISO 22196:2011 standard protocol. In brief, bacterial suspensions were prepared by culturing each strain overnight at 34°C in Luria-Bertani (LB) broth under aerobic conditions, followed by dilution in 1/500 nutrient broth (NB) to achieve a final concentration of 1 × 10^6^ colony-forming units (CFU) mL^−1^. Test specimens were aseptically positioned in sterile petri dishes, followed by the application of 100 μL inoculum onto each sample surface to ensure complete coverage without spillage beyond the specimen edges. Following inoculation, samples were incubated for 24 h at 34°C under controlled environmental conditions (≥90% relative humidity). Pristine disks served as negative controls throughout the experimental protocol.

#### 2.5.2 Live/dead bacterial staining

To evaluate adherent bacterial viability, the test specimens were subjected to a live/dead fluorescence viability assay. Following a 24 h incubation period under standardized conditions, samples were gently rinsed three times with sterile phosphate-buffered saline (PBS, pH 7.4) to remove non-adherent cells. Bacterial viability staining was performed using the Live/Dead^®^ BacLight™ Bacterial Viability Kit (EX3000, Solarbio, Beijing, China), with 1 μL mL^−1^ NucGreen™ (nucleic acid staining for live cells) and 2 μL mL^−1^ Ethidium Homodimer-III (EthD-III, membrane-impermeant dye for dead cells) dissolved in 0.9% (w/v) NaCl solution. After 15 min of incubation in darkness at room temperature, the dyed surfaces were analyzed using an Axio Observer Inverted microscope (Carl Zeiss Microscopy, Germany).

#### 2.5.3 Characterization of adherent bacteria with SEM

The disks retrieved from incubation (24 h) were characterized using SEM to visualize adherent bacteria and biofilm. Briefly, disks were fixed with 2.5% glutaraldehyde (G105907, Aladdin) for 30 min and sequentially dehydrated with 30%, 50%, 70%, 90%, and 100% ethanol for 10 min each, followed by chemical drying overnight with pure Hexamethyldisilazane (H106018, Aladdin) in a fume hood. After sputter coating with carbon, images were captured by a (JEOL JSM-7000F, United States) at 15 kV under high vacuum conditions.

#### 2.5.4 Quantification of viable bacterial colonies via CFU assays

To evaluate the viable planktonic and adherent bacteria after co-incubate with samples for 24 h, the inoculum and samples were collected in 1.5 mL PBS and were subjected to sequential sonication (5 min) and vortexing (1 min) to dislodge adherent bacteria from the surfaces. The resulting eluents were serially diluted 10-fold in sterile PBS. Aliquots (100 μL) of each dilution were aseptically plated onto sterile Petri dishes, followed by the addition of 15 mL of molten Plate Count Agar (MilliporeSigma, 70189) at 45°C. The agar-bacteria mixtures were gently swirled to ensure homogeneous dispersion and allowed to solidify. Plates were incubated aerobically at 34°C ± 0.5°C for 24 h under controlled humidity (≥90% RH). Post-incubation, CFUs were enumerated per plate. Antibacterial activity (%) was calculated using Equation 1:

$$Antibacterial\;rate\;\left(\%\right)=\frac{{CFU}_{Control}-{CFU}_{treated}}{{CFU}_{Control}}\times 100\%$$ (1)

To determine the sustained antibacterial activity of modified surfaces, the samples were incubated with PBS for 4 days, with the PBS solution replenished daily to simulate physiological fluid exchange. Following this conditioning period, the antibacterial efficacy of the surfaces was quantified using the same experimental protocol as described above.

#### 2.5.5 Implant-related infection rat models

To evaluate the anti-infective and osseointegration potential of surface-modified implants, an established rat model of *S aureus*-induced implant-associated osteomyelitis was employed. Thirty-two male SD rats (250–300 g) were randomly allocated into five experimental groups with 4 rats for each experimental test under protocols approved by the Institutional Animal Care and Use Committee of the Chongqing Institute for Food and Drug Control. Following general anesthesia induced by intraperitoneal injection of 1% sodium pentobarbital (50 mg kg^−1^), femur articular surfaces were surgically exposed. A bone defect (diameter: 1.5 mm; depth: 10 mm) was created in the metaphyseal region from articular cartilage using low-speed rotary drilling under continuous sterile saline irrigation. 10 μL of *S*. *aureus* suspension (1 × 10^6^ CFU mL^−1^ in PBS) was inoculated into the osseous channel via a precision micropipette. Test implants were then aseptically inserted into the contaminated defects. The incision was closed with 5–0 polyglycolic acid sutures (Vicryl) and the rats were allowed to move freely in the cages.

#### 2.5.6 *In vivo* antibacterial efficacy evaluation

Rats with surgically implanted samples were euthanized at a 3-day postoperative timepoint to assess acute infection control. Post-explantation, the retrieved implant rods were subjected to two complementary bacteriological analyses. First, implants were gently rolled onto sterile agar plates under aseptic conditions to transfer adherent surface bacteria, followed by 24 h aerobic incubation at 34°C. Concurrently, residual implants were immersed in 1.5 mL of PBS and mechanically agitated via ultrasonication (5 min) and vortex mixing (1 min) to dislodge microorganisms. The resulting bacterial suspensions were serially diluted, and 50 µL aliquots were plated onto nutrient agar for CFU enumeration. After 24 h incubation at 34°C, bacterial colonies were imaged and quantified using standardized counting protocols to determine the antibacterial performance of each experimental group.

### 2.6 Peri-implant bone regeneration evaluation

#### 2.6.1 Micro-CT scanning to evaluate bone regeneration

Following a 4-week implantation, rats from Ti64, PDA and PDA@Li 800-Cu 10 were euthanized via anesthetic overdose, and femurs containing titanium implants were surgically excised. The harvested specimens were fixed in 4% paraformaldehyde (PFA) for 48 h. Micro-CT was performed using an NMC-200 system (PINGSENG SCIENTIFIC, China) operating with an isotropic voxel resolution of 35 μm to assess peri-implant bone formation. A concentric region of interest (ROI) spanning 500 μm radially from the implant surface was defined to standardize morphometric analysis. Key trabecular parameters, including bone volume, bone volume fraction (BV/TV, %) and trabecular spacing (Tb.Sp, mm), were quantified.

#### 2.6.2 Histological evaluation

Following micro-CT scanning, the fixed femoral specimens were subjected to decalcification in 10% (w/v) ethylenediaminetetraacetic acid (EDTA) disodium solution for 21 days. Titanium implants were subsequently carefully extracted from the decalcified femurs using minimal mechanical disruption to avoid compromising the surrounding bone-implant interface. The specimens were dehydrated through a graded ethanol series (70%–100%), cleared in n-butyl alcohol, and embedded in paraffin. Transverse histological sections (5 μm thickness) perpendicular to the longitudinal axis of the implant were prepared using a Leica SP1600 saw microtome (Leica Microsystems, Hamburg, Germany). Hematoxylin and eosin (HE) staining was performed to evaluate osseous morphology and peri-implant tissue remodeling. Pictures were captured using a Leica DM6 B LED Microscope equipped with LAS X imaging software (Leica Microsystems).

### 2.7 Statistical analysis

All quantitative data are presented as mean ± standard error of the mean derived from repeated independent experiments. Statistical analyses were conducted using GraphPad Prism software (version 9.0, GraphPad Inc., USA). Intergroup comparisons were evaluated using a two-tailed unpaired Student’s t-test for two-group analyses, whereas one-way analysis of variance (ANOVA) with Tukey’s *post hoc* test was applied for multi-group comparisons. A significance threshold of *p* < 0.05 was established for all statistical evaluations, with asterisk notation (*) denoting statistically significant differences in graphical representations.

## 3 Results and discussion

### 3.1 Characterization of surface topography and components

Titanium and its alloys have been clinically validated as standard materials for bone implant fabrication, demonstrating long-term (>10 years) success rates exceeding 95% in follow-up studies (Premkumar et al., 2021; Stauss et al., 2024). To further advance implant performance, ongoing research focuses on optimizing substrate physicochemical properties such as material composition, mechanical strength, as well as surface topography and chemics to enhance osseointegration and mitigate infection risks (Kurtz et al., 2012; Lin et al., 2017; Australian Orthopaedic Association National Joint Replacement Registry, 2018; Croes et al., 2018; Canadian Institute for Health Information, 2019). Surface modification strategies, in particular, exert the most direct influence on peri-implant bone remodeling and cellular behavior. Established surface modification techniques, including plasma spraying, electrochemical anodization and bioactive polymer coatings, have demonstrated enhanced osseointegration and/or antimicrobial efficacy (Sharath Kumar et al., 2023). Addressing the technical challenges associated with uniform modification of complex three-dimensional (3D) geometries, polydopamine (PDA) coatings have emerged as a versatile strategy. PDA not only enhances cellular interactions by compensating for substrate biocompatibility limitations but also synergistically improves osteoconductivity and antibacterial activity through the controlled incorporation of bioactive agents, such as growth factors, metal ions (e.g., Ag^+^, Zn^2+^), and antibiotics (Lyu et al., 2022; Mahnavi et al., 2023).

**FIGURE 1:**
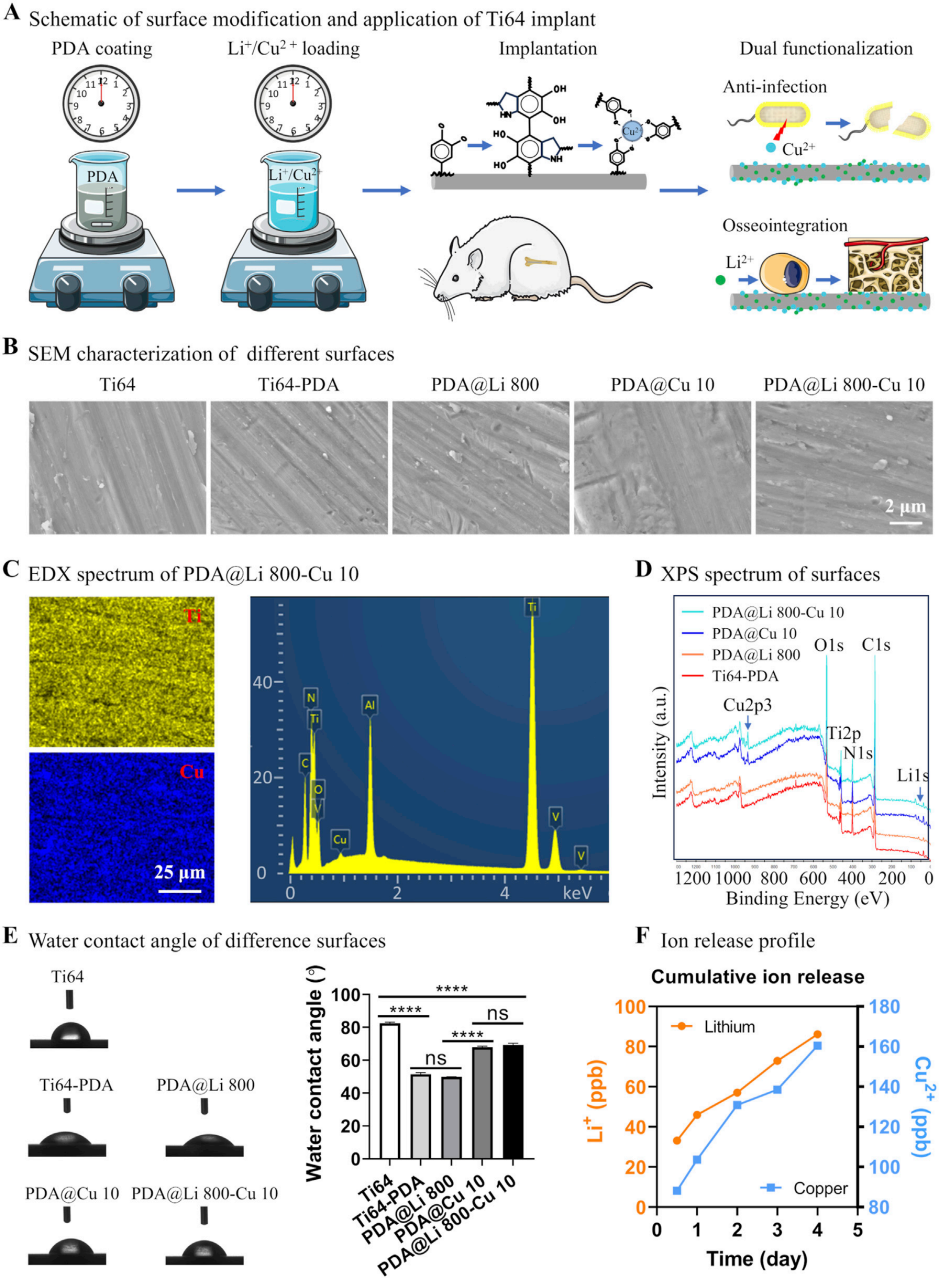
Schematic and physicochemical characterization of titanium surface modification via a mussel-inspired ion immobilization strategy. **(A)** A scalable dip-coating technique to deposit a conformal PDA thin film on Ti-6Al-4V substrates, illustrating its integration into bone implant surface engineering. **(B)** SEM characterization of the nanoscale topography and uniformity of the PDA-coated surface following Li^+^/Cu^2+^ ion immobilization (scale bar: 2 μm). **(C)** EDX elemental mapping confirmed the successful incorporation and homogeneous distribution of Cu across the functionalized surface (scale bar: 25 μm). **(D)** XPS analysis validated the chemical composition of the modified surfaces. **(E)** Representative water contact angle images of different sufaces and measured results. **(F)** Cu^2+^ and Li^+^ release profiles of PDA@Li 800-Cu 10 over 4 days incubation in PBS.

Figure 1A demonstrates the fabrication of a PDA-modified implant via a two-step methodology. Initially, cleaned implants were immersed in the Tris-HCl buffer (pH 8.5) with dopamine dissolved. Subsequently, the PDA-coated surfaces were functionalized with metal ions (Cu^2+^ and Li^+^) through ion coordination. The incorporation of Cu^2+^ imparts bactericidal properties to the implant, while Li^+^ immobilization enhances osteogenic activity. SEM analysis revealed that the PDA coating, even with metal ion integration, did not significantly alter surface morphology, only minor scratches originating from silicon paper polishing were observed (Figure 1B). EDX confirmed successful Cu^2+^ loading, as evidenced by elemental mapping and spectral peaks (Figure 1C). However, Li^+^ detection via EDX was hindered by its intrinsic limitations, including low atomic number, weak X-ray emission, and detector design constraints. To address this, XPS was employed for comprehensive surface analysis, Distinct Cu 2p peaks were detected on both PDA@Cu 10 and PDA@Li 800-Cu 10 surfaces, validating Cu^2+^ incorporation (Figure 1D). In contrast, Li^+^ signals were attenuated due to the low sensitivity of the Li 1s photoelectron peak, yielding only weak spectral signatures for PDA@Li 800 and PDA@Li 800-Cu 10 surfaces.

As shown in Figure 1E, polydopamine (PDA) coating significantly enhanced the hydrophilicity of Ti64 surfaces, reducing the water contact angle (WCA) from 83° (polished control) to 51°. Subsequent lithium (Li^+^) incorporation maintained this hydrophilic character (WCA = 50°), consistent with prior reports of Li^+^ functionalization (Zhang Q. et al., 2024). In contrast, copper (Cu^2+^)-incorporated surfaces (PDA@Cu 10 and PDA@Li 800-Cu 10) exhibited a distinct hydrophobic shift, increasing WCA to 67°–70° (*p* < 0.0001 vs PDA). While this trend contradicts the typical wettability enhancement observed with Sr^2+^ or Li^+^ loading (Chung et al., 2024; Zhang Q. et al., 2024), it aligns with studies reporting elevated WCAs for Cu- or Zn-functionalized PDA coatings (Wang et al., 2017; Wang et al., 2019). This divergence suggests that cation-specific interfacial interactions, rather than PDA’s intrinsic hydrophilicity, dominate surface wettability. The molecular basis for this cation-dependent wettability requires deeper investigation.

As evidenced by the ICP-OES results in Figure 1F, both Cu^2+^ and Li^+^ exhibited continuous release profiles throughout the 4-day observation period, with Cu^2+^ demonstrating significantly higher cumulative release compared to Li^+^. At the 12-h timepoint, Li^+^ concentrations reached ∼35 parts per billion (ppb), whereas Cu^2+^ levels peaked at ∼90 ppb, highlighting faster initial elution kinetics for Cu^2+^. This aligns with prior reports of PDA-mediated Cu^2+^ delivery systems, where the rapid early-stage release was attributed to weak coordination between Cu^2+^ and surface-exposed catechol groups (Wang et al., 2017). Conversely, Li^+^’s slower, sustained release profile—presumed to arise from stronger interactions with amine residues deeper within the PDA matrix—mirrors patterns observed in plasma electrolytic oxidation (PEO)-treated surfaces (Peng et al., 2021; Qiu et al., 2022). However, the PDA-based approach offers distinct advantages over PEO, including milder processing conditions (aqueous, room temperature) and reduced substrate degradation risks, making it more suitable for clinical-grade titanium implants.

The differential release kinetics likely underpin the observed therapeutic synergy: rapid Cu^2+^ elution suppresses bacterial colonization during the critical postoperative period, while sustained Li^+^ delivery promotes long-term osteogenesis. These findings validate PDA’s versatility in modulating ion release rates through tailored coordination chemistry, even in dual-ion systems. Future studies should explore advanced PDA architectures (e.g., layered or crosslinked networks) to further decouple release timelines for enhanced spatiotemporal control.

### 3.2 Effects of Li^+^ and Cu^2+^ incorporated surfaces on MC3T3-E1 proliferation and osteogenesis

#### 3.2.1 Effects of Li^+^ incorporated surfaces on MC3T3-E1 proliferation and osteogenesis

**FIGURE 2:**
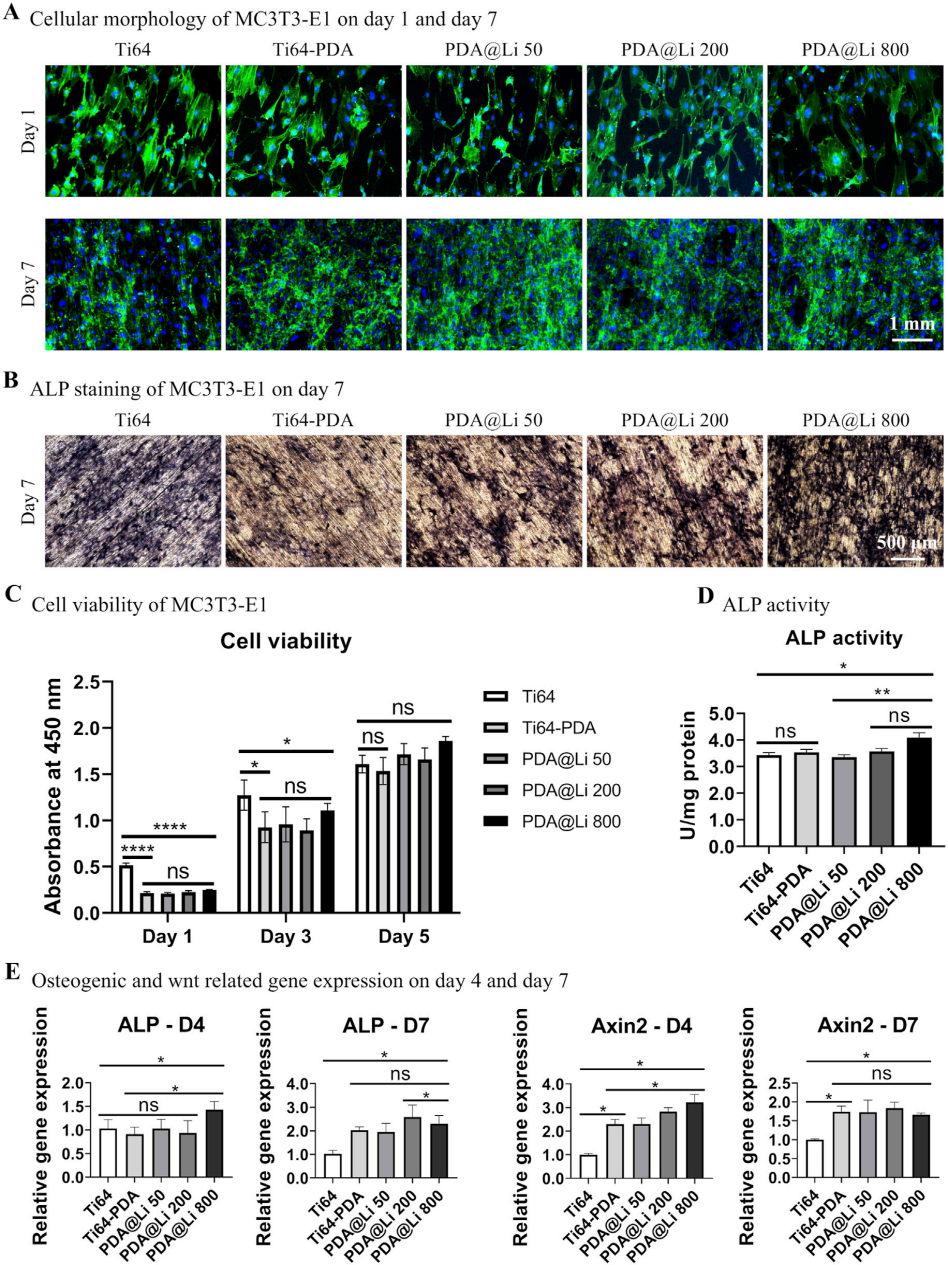
Characterization of the effect of Li^+^-incorporated surfaces on MC3T3-E1 morphogenesis, proliferation, and osteogenic differentiation. **(A)** Representative fluorescence images of F-actin cytoskeletal organization (phalloidin, green) and nuclei (Hoechst 33258, blue) in MC3T3-E1 cells cultured on different surfaces at 1 and 7 days post-seeding. Scale bar: 1 mm. **(B)** ALP activity staining after 7 days of culture. Scale bar: 500 μm. **(C)** Quantitative cell viability was assessed via CCK-8 assay on days 1, 3 and 5. *p < 0.05, ****p < 0.0001 (n = 3). **(D)** Normalized ALP activity quantification at day 7. *p < 0.05¸**p < 0.01 (n = 3). **(E)** qRT-PCR analysis of *ALP* and *Axin2* mRNA expression at days 4 and 7. *p < 0.05 (n = 3).

The biocompatibility and osteogenic differentiation capacity of MC3T3-E1 pre-osteoblasts cultured on Ti-6Al-4V surfaces with varying Li^+^ concentrations were systematically evaluated (Figure 2). Cell morphology and adhesion behavior were first determined by Phalloidin/DAPI staining after culturing for 1 and 7 days. Overall, cells on polished Ti-6Al-4V surfaces and the DA-treated surface (with/without Li incorporation) exhibited rounded, irregular morphologies regardless of Li^+^ concentration (Figure 2A). Despite initial morphology discrepancies, all surfaces supported robust cellular proliferation following 7 days of culture, with confluent cell layers observed. CCK8 assays corroborated these findings: cellular activity increased temporally across groups, though cells on DA-coated surfaces showed significantly lower overall bioactivity at day 1 (*p* < 0.05). By day 7, proliferation disparities diminished, with comparable viability among all groups (Figure 2C).

To explore the effect of different treated surfaces on bone regeneration, an *in vitro* study was performed to investigate the osteogenic differentiation capacity of MC3T3-E1 on different surfaces. ALP staining and quantification demonstrated concentration-dependent osteogenic enhancement by Li^+^ incorporation (Figures 2B,D). DA coating alone did not elevate ALP activity directly, but Li^+^ immobilization at 800 mM significantly increased ALP activity compared to polished controls (p < 0.05) and DA-only surfaces (*p* < 0.05) by day 7 of culture.

RT-PCR further revealed temporal representative osteogenic differentiation-related and wnt activation-related gene regulation patterns (Figure 2E). At day 4, 800 mM Li-treated surfaces markedly upregulated *ALP* expression in comparison to polished control and other lower-concentration Li^+^-treated surfaces. In terms of the *Axin2* (a Wnt/β-catenin marker), DA treatment significantly promoted the gene expression and Li immobilization further synergistically upregulated its expression. By day 7, *ALP* expression on DA-coated surfaces (regardless of Li^+^) surpassed polished controls (*p* < 0.05), though the Li^+^-induced synergistic effect on *Axin2* diminished compared with that of D4, leaving DA coatings alone still significantly elevated that compared to polished control.

#### 3.2.2 Effects of Cu^2+^ incorporated surfaces on MC3T3-E1 proliferation and osteogenesis

**FIGURE 3:**
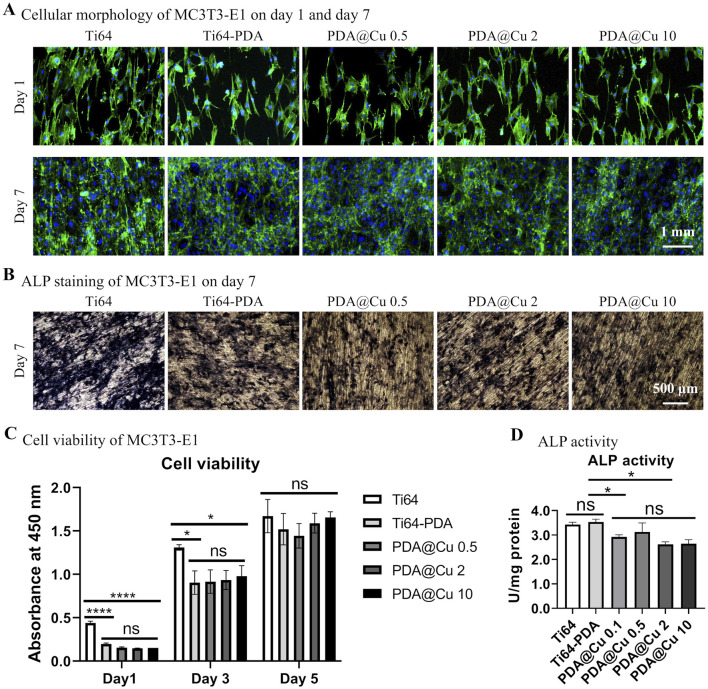
Characterization of cell morphology, proliferation and osteogenic differentiation of MC3T3-E1 on Cu^2+^-incorporated surfaces. **(A)** Fluorescence microscopy images of cytoskeletal architecture via phalloidin staining (F-actin, red) and nuclei (Hoechst 33258, blue) after 1 and 7 days of culture. Scale bars: 1 mm. **(B)** ALP staining at day 7. Scale bar: 500 μm. **(C)** Quantification of cell proliferation by CCK-8 assay on Cu-doped coatings over time. *p < 0.05, ****p < 0.0001 (n = 3). **(D)** Normalized ALP activity corroborates the staining results relative to unmodified groups at day 7. *p < 0.05 (n = 3).

Pre-osteoblast proliferation and osteogenic differentiation were further investigated. As shown in Figure 3, Cu^2+^ concentrations of 0.1, 0.5, 2, and 10 mM exhibited no significant alteration in cellular morphology or proliferation pattern compared to polished control on day 1, except for lower cell density for DA-treated surfaces (with/without Cu). By day 7, all substrate surfaces were fully confluent with cells, and cellular viability (assessed via CCK-8 assay) showed no statistically significant differences between groups, mirroring the proliferation trends observed on lithium (Li)-treated surfaces. In contrast, ALP staining and quantitative analysis revealed a concentration-dependent inhibition effect of Cu^2+^ incorporation on osteogenic differentiation. These results suggest that while Cu^2+^ incorporation does not compromise short-term cell adhesion or proliferation, it significantly suppresses osteogenic commitment in MC3T3-E1 cells.

The dual role of polydopamine (PDA) coatings—as a versatile surface modification tool and a transient modulator of cellular behavior—presents both challenges and opportunities for bone tissue engineering applications. While early studies predominantly focused on PDA’s substrate-independent adhesion and biofunctionalization capabilities (Yu et al., 2022), recent findings, including ours, highlight its paradoxical effects on cell viability and differentiation. Our results demonstrate that PDA coatings initially suppress MC3T3-E1 adhesion and proliferation (day 1), aligning with reports of reduced viability in diverse cell types (HUVECs, HFFs, A549) across substrates (glass, PDMS, Ti6Al4V) (Wang et al., 2017; Yu et al., 2022). However, this inhibitory effect is transient, as cellular activity equilibrates across all groups by day 7, suggesting that PDA’s hydrophobicity or residual oxidative byproducts may impede early adhesion without compromising long-term biocompatibility. Critically, this temporal recovery aligns with clinical timelines for implant osseointegration, where short-term inflammatory responses often precede sustained tissue remodeling.

The integration of bioactive elements like Li^+^ into PDA coatings offers a strategic solution to counterbalance these transient drawbacks while amplifying therapeutic outcomes. Our data reveal that Li^+^ incorporation at 800 mM synergistically enhances osteogenic differentiation, as evidenced by significant *ALP* and *Axin2* upregulation at day 4 (Figure 2E). This early Wnt/β-catenin activation (via Axin2) likely primes cells for accelerated matrix mineralization, a hypothesis supported by prior studies linking Li^+^-induced GSK-3β inhibition to osteoblast commitment (Galli et al., 2012). Notably, PDA’s catechol-rich structure facilitates controlled Li^+^ release, mitigating cytotoxic bursts associated with direct ion doping. This dual functionality—surface passivation and ion reservoir—positions PDA as a uniquely adaptable platform for spatiotemporal delivery of osteoinductive agents.

The intrinsic bioactivity of polydopamine (PDA) coatings necessitates critical evaluation. While Li-free PDA coatings enhanced osteogenic gene expression (*ALP*, *Axin2*) by day 7—consistent with prior studies demonstrating PDA’s pro-osteogenic properties (Li et al., 2019; Zhong et al., 2024)—the underlying mechanisms involve multifaceted interactions. Specifically, dopamine-related functional groups on PDA surfaces activate the dopamine D1 receptor (D1R), triggering downstream signaling cascades that upregulate osteogenic transcription factors (Zhu et al., 2022). Concurrently, PDA’s mineral-rich interface facilitates hydroxyapatite nucleation, mimicking the biochemical and topographical cues of the native osteogenic niche to direct mesenchymal stem cell (MSC) differentiation (Zhu et al., 2022; Wang et al., 2024). These dual mechanisms synergistically enhance bone regeneration, as evidenced by *in vitro* and *in vivo* models (Li et al., 2019; Kong et al., 2020; Zhong et al., 2024). However, the precise relationship between PDA’s physicochemical properties (e.g., surface roughness, charge density) and intracellular signaling pathways (e.g., D1R-mediated cAMP/PKA/CREB activation) remains poorly defined, warranting further investigation into structure-function correlations. The observed Axin2 upregulation—a hallmark transcriptional target of canonical Wnt/β-catenin signaling—strongly suggests that Li^+^ incorporation activates this pathway via GSK-3β inhibition, consistent with prior reports (Beurel et al., 2004; O'Brien and Klein, 2009; Abdul et al., 2018; Duda et al., 2020). While direct visualization of β-catenin nuclear translocation or GSK-3β phosphorylation status would further corroborate this mechanism, such analyses fall outside the translational scope of this study and will be pursued in subsequent work.

The transient bioactivity of PDA coatings—characterized by early-stage adhesion modulation followed by sustained osteogenic promotion—represents a tunable design feature rather than a limitation. By integrating PDA’s dynamic interfacial adaptability with programmable bioactive ion release (e.g., Li^+^, Sr^2+^), next-generation implants can achieve spatiotemporal regulation of bone healing, transcending the static functionality of traditional biomaterials.

### 3.3 Antibacterial capacity of Cu immobilized surface

**FIGURE 4:**
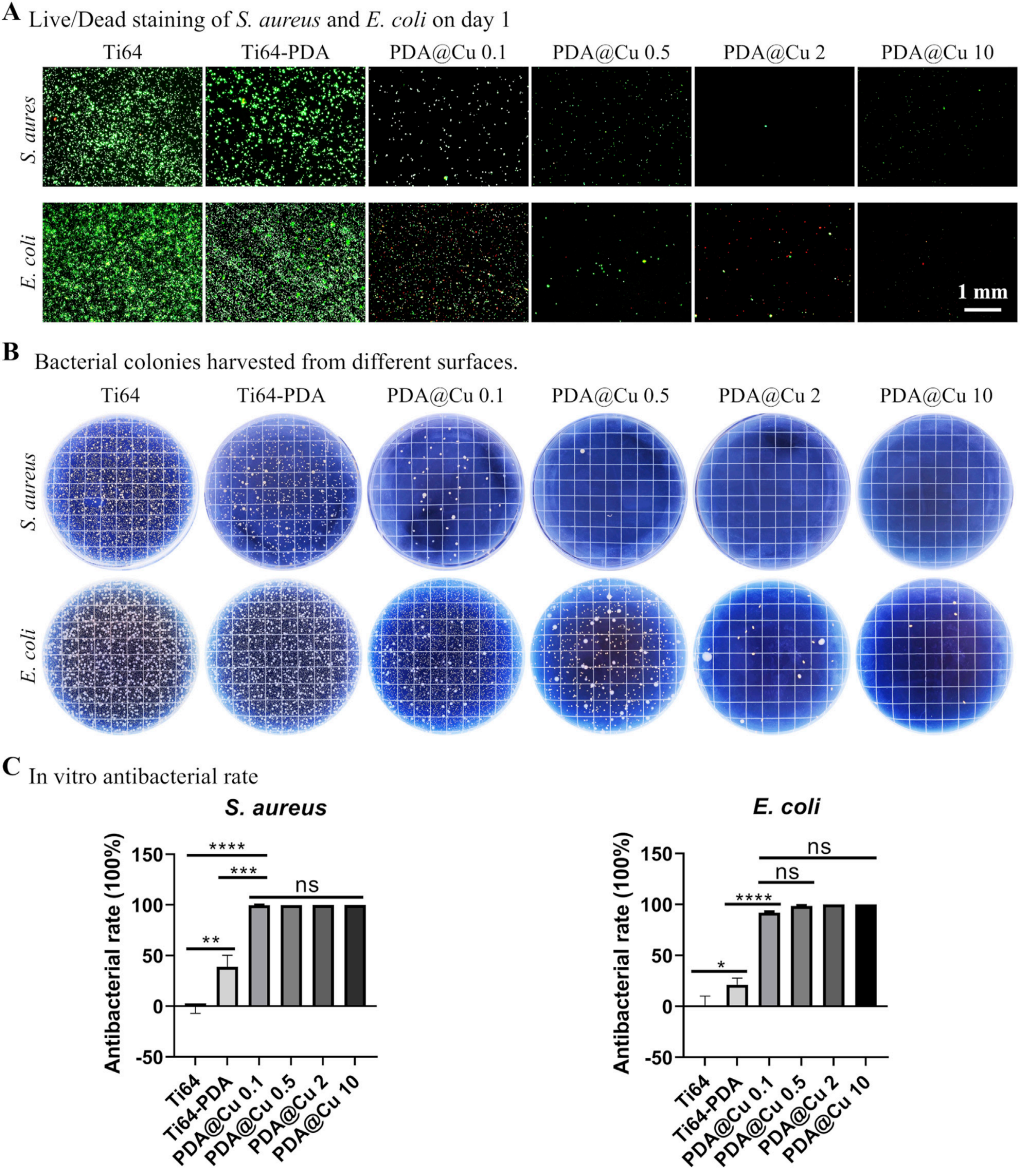
Antibacterial efficacy of copper-incorporated functional surfaces. **(A)** Fluorescence micrographs of live/dead-stained *Staphylococcus aureus* and *E. coli* colonies cultured on modified surfaces for 24 h, with viable bacteria (green) and dead bacteria (red) visualized. Scale bars: 1 mm. **(B)** Representative photographic documentation of bacterial colonies harvested from surfaces. **(C)** Quantitative assessment of antibacterial efficacy, calculated via CFU reduction rates. *p < 0.05, **p < 0.01, ***p < 0.001, ****p < 0.0001 (n = 3).

The antibacterial efficacy of Cu^2+^ functionalized surface was systematically evaluated against Gram-negative (*E. coli*) and Gram-positive (*S. aureus*) pathogens, as illustrated in Figure 4A. Live/dead fluorescence staining revealed stark contrasts in bacterial viability across treatment groups. Polished Ti64 surfaces exhibited negligible intrinsic antibacterial activity, with dense green fluorescence (SYTO 9- stained viable cells) observed for both *E coli* and *S. aureus*. In contrast, DA-coated surfaces demonstrated modest antibacterial effects (about 20%–30% induction in viability vs. Ti64). Consistent with prior reports of PDA’s weak bacteriostatic properties (Su et al., 2016; Wang et al., 2019; Yang et al., 2023). Strikingly, Cu^2+^-incorporated surfaces exhibited concentration-dependent bactericidal activity: 0.1 mM Cu-treated surface reduced viable bacteria by > 90%, while higher concentrations (≥0.5 mM) achieved near-complete eradication (Figure 4B and 4C). Further SEM characterization (Supplementary Figure S1) corroborated the live/dead staining results, demonstrating a dose-dependent antibacterial efficacy of copper (Cu)-functionalized surfaces. On Ti64 and PDA-only controls, dense bacterial colonization was observed, with intact cellular morphologies. In contrast, PDA@Cu 0.1 and PDA@Cu 0.5 surfaces exhibited sparse, isolated bacterial cells. Remarkably, PDA@Cu 2 and PDA@Cu 10 surfaces showed no detectable bacterial colonization, confirming complete bactericidal activity at higher Cu loadings.

CFU assays corroborated these findings, showing a log-scale reduction in bacterial load proportional to treated Cu^2+^ concentration (*p < 0.001*) with 10 mM Cu^2+^ yielding undetectable CFUs for both strains. This concentration-dependent bactericidal effect was in line with previous reports modifying titanium surfaces with copper ions (He et al., 2014; Wu et al., 2014; Wang et al., 2017), indicating its potential application in bone implant fabrication, however, how to correlate the *in vitro* results to *in vivo* precisely is still challenging. Further study should comprehensively investigate the *in vitro* anti-bacterial to broader species especially the antibiotic ones and *in vivo* anti-infection capacity.

### 3.4 Combined Li and Cu incorporation modulates MC3T3-E1 proliferation and osteogenesis

**FIGURE 5:**
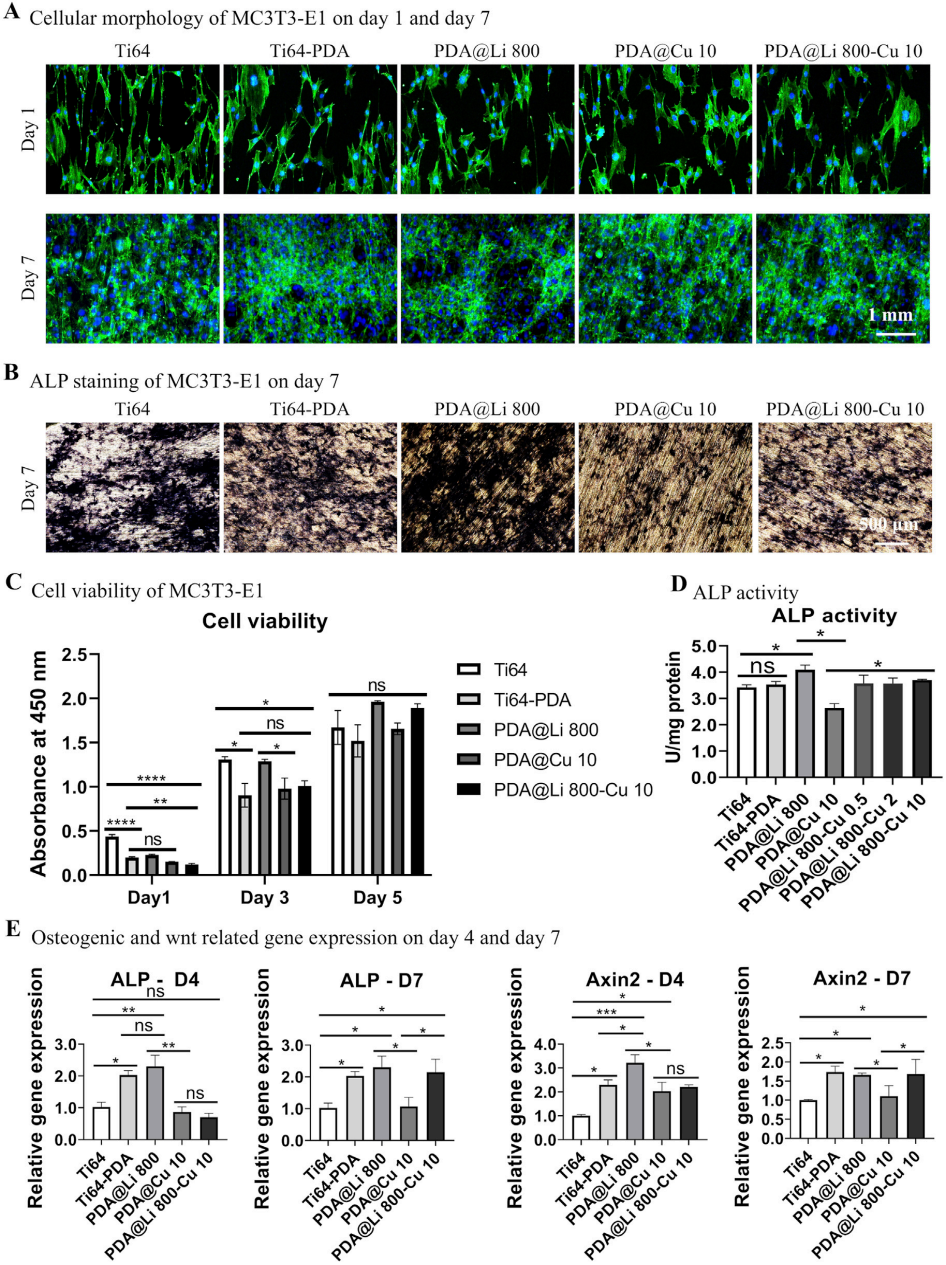
Characterization of the effect of dual-functional Li^+^/Cu^2+^-incorporated coatings on MC3T3-E1 morphogenesis, proliferation, and osteogenic differentiation. **(A)** Representative fluorescence images of F-actin cytoskeletal organization (phalloidin, green) and nuclei (Hoechst 33258, blue) in MC3T3-E1 cells cultured on different surfaces at 1 and 7 days post-seeding. Scale bar: 1 mm. **(B)** ALP activity staining after 7 days of culture. Scale bar: 500 μm. **(C)** Quantitative cell viability was assessed via CCK-8 assay on days 1, 3 and 5. *p < 0.05, ****p < 0.0001 (n = 3). **(D)** Normalized ALP activity quantification (absorbance at 405 nm) at day 7. *p < 0.05¸**p < 0.01 (n = 3). **(E)** qRT-PCR analysis of *ALP* and *Axin2* mRNA expression at days 4 and 7. *p < 0.05 (n = 3).

To evaluate the synergistic potential of Li^+^ and Cu^2+^ co-delivery, MC3T3-E1 proliferation and differentiation were assessed on dual–ion functionalized Ti64 surfaces. Cellular morphology remained consistent across all groups, with no observable cytotoxicity–a finding corroborated by live/dead staining (Figure 5A). Proliferation kinetics, quantified via CCK-8 assay, revealed that all surfaces sustained cell growth over 7 days, achieving comparable viability (Figure 5C). Notably, the 800 mM Li^+^-modified surface exhibited accelerated proliferation by day 5, though statistical significance was not reached by day 7, consistent with prior reports of Li^+^-enhanced mitogenesis via Wnt/β-catenin activation (Li et al., 2017).

Osteogenic differentiation, however, exhibited stark ion-dependent contrasts (Figures 5B,D). ALP staining and quantification revealed that 800 mM Li^+^ treated surfaces significantly augmented early osteogenesis whereas 10 mM Cu^2+^ of that suppressed the ALP activity level. Strikingly, dual Li^+^/Cu^2+^ incorporation rescued ALP activity to levels comparable to dopamine-coated controls, suggesting Li counteracts Cu^2+^-mediated osteogenic inhibition. Gene expression profiling further elucidated these dynamics (Figure 5E). At day 4, 800 mM Li^+^ upregulated *ALP* and *Axin2*, confirming Li^+^ drive Wnt pathway activation. In contrast, 10 mM Cu^2+^ had no early effect on osteogenic transcripts by suppressing *ALP* and *Axin2* by day 4 and day 7, aligning with Cu^2+^ induced ROS–medicated Wnt pathway dysregulation (Zhang et al., 2018). Dual Li^+^/Cu^2+^ surfaces restored *ALP* and *Axin2* expression to DA–treated levels by day 7, indicating Li^+^‘s dominance in sustaining osteogenic transcription despite Cu^2+^ co-presence.

### 3.5 *In vitro* and *in vivo* antibacterial efficiency of combined Li and Cu incorporated surface

**FIGURE 6:**
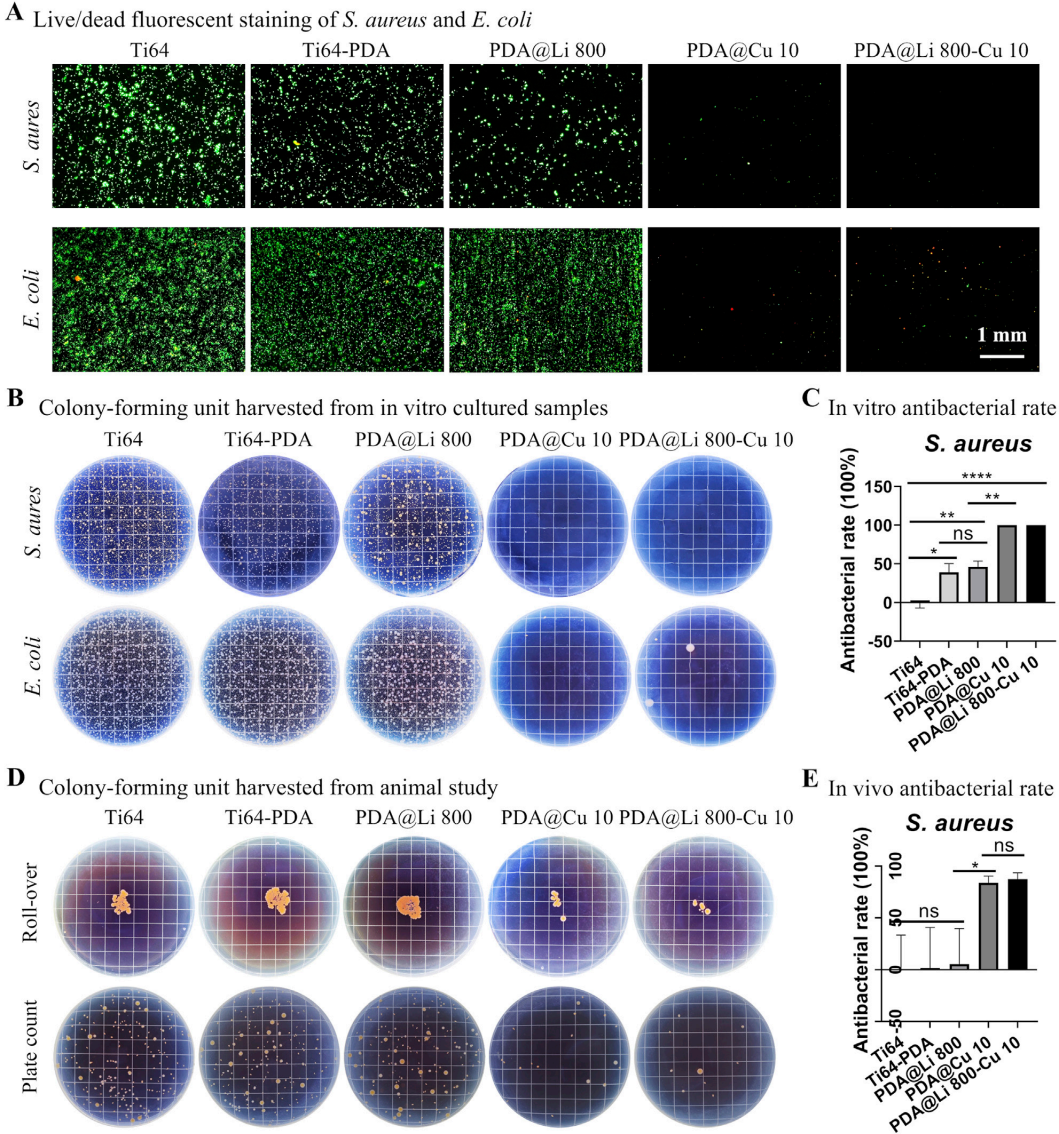
*In vitro* and *in vivo* antibacterial evaluation of dual-functional Li^+^/Cu^2+^-incorporated coatings. **(A)** Live/dead fluorescent staining of *Staphylococcus aureus* and *E. coli* cultured for 24 h on modified surfaces (green: live; red: dead). Scale bar: 1 mm. **(B)** Representative CFU harvested from surfaces after 24 h of culture. **(C)** *In vitro* quantitative analysis for dual-ion-modified surfaces. *p < 0.05, **p < 0.01, ****p < 0.0001 (n = 4). **(D)** Surface imprints and spread plate analyses of 3 days implanted samples. **(E)** Quantification of *in vivo* bacterial retention. *p < 0. 05 (n = 4).

The antimicrobial potential of Cu^2+^ and Li^+^-functionalized Ti64 surfaces was systematically evaluated against *S. aureus* and *E. coli* pathogens (Figure 6). *In vitro* live/dead staining revealed extensive bacterial colonization on polished Ti64, whereas DA-coated surfaces exhibited moderate adhesion reduction (38.3% vs Ti64, *p* < 0.05) (Figure 6A). Strikingly, Cu^2+^-incorporated surfaces (PDA@Cu 10 and PDA@Li 800-Cu 10) demonstrated near-complete bacterial eradication (>99% reduction in viable cells, *p* < 0.001), consistent with Cu^2+^’s capacity to disrupt membrane integrity via lipid peroxidation and ROS generation. While Li^+^-modified surfaces (PDA@Li 800) exhibited a modest reduction in viable bacteria (∼46%), this effect aligned with the baseline antibacterial activity of polydopamine (Ti64-PDA: 38.3%, *p* > 0.05). This suggests that Li^+^ incorporation does not enhance intrinsic antibacterial activity, as the observed reduction likely stems from PDA’s inherent catechol-mediated bacteriostatic properties rather than Li^+^-specific mechanisms. The SEM images confirmed the live/dead staining, supporting the bacterial efficacy of Cu-incorported surfaces (PDA@Cu 10 and PDA@Li 800-Cu 10) (Supplementary Figure S2). Quantitative CFU assays further corroborated these findings: Cu^2^-functionalized surfaces reduced *S. aureus* colonization by more than 95% compared to polished Ti64 (*p* < 0.001) (Figures 6B,C).

Since current evidence suggests that the highest risk of implant-associated infection occurs during the early perioperative period, when bacterial contamination from endogenous flora or exogenous surgical sources is most likely to overwhelm host defenses (Kapadia et al., 2016; Arciola et al., 2018), This aligns with the “race for the surface” paradigm, wherein bacterial colonization and host cell integration compete for dominance at the implant-tissue interface (Shiels et al., 2020). Therefore the antibacterial ability sustained for a few days would be beneficial for the infection control of implant lifecycle. Supplementary Figure S3 demonstrated that after immersing Cu-loaded titanium samples in PBS for 4 days (simulating early postoperative conditions), the samples sustained bactericidal activity, evidenced by the comparable antibacterial rate to fresh samples (Figure 4C). This indicates that the samples remain effective during the critical window of infection risk over a few days.

Implantation in a rat model mirrored *in vitro* trends (Figure 6D). After 72 h of implantation, polished Ti64, Ti64-PDA and PDA@Li 800 samples exhibited rampant bacterial proliferation, whereas PDA@Cu10 and PDA@Li 800-Cu 10 coatings reduced bacterial loads by >80% (*p* < 0.01) (Figure 6E). The retained antibacterial efficacy *in vivo* suggests stable Cu^2+^ release kinetics, a phenomenon attributed to PDA’s catechol-mediated ion chelation, indicating their anti-infection potential.

### 3.6 Animal studies evaluate the peri-implant bone regeneration

**FIGURE 7:**
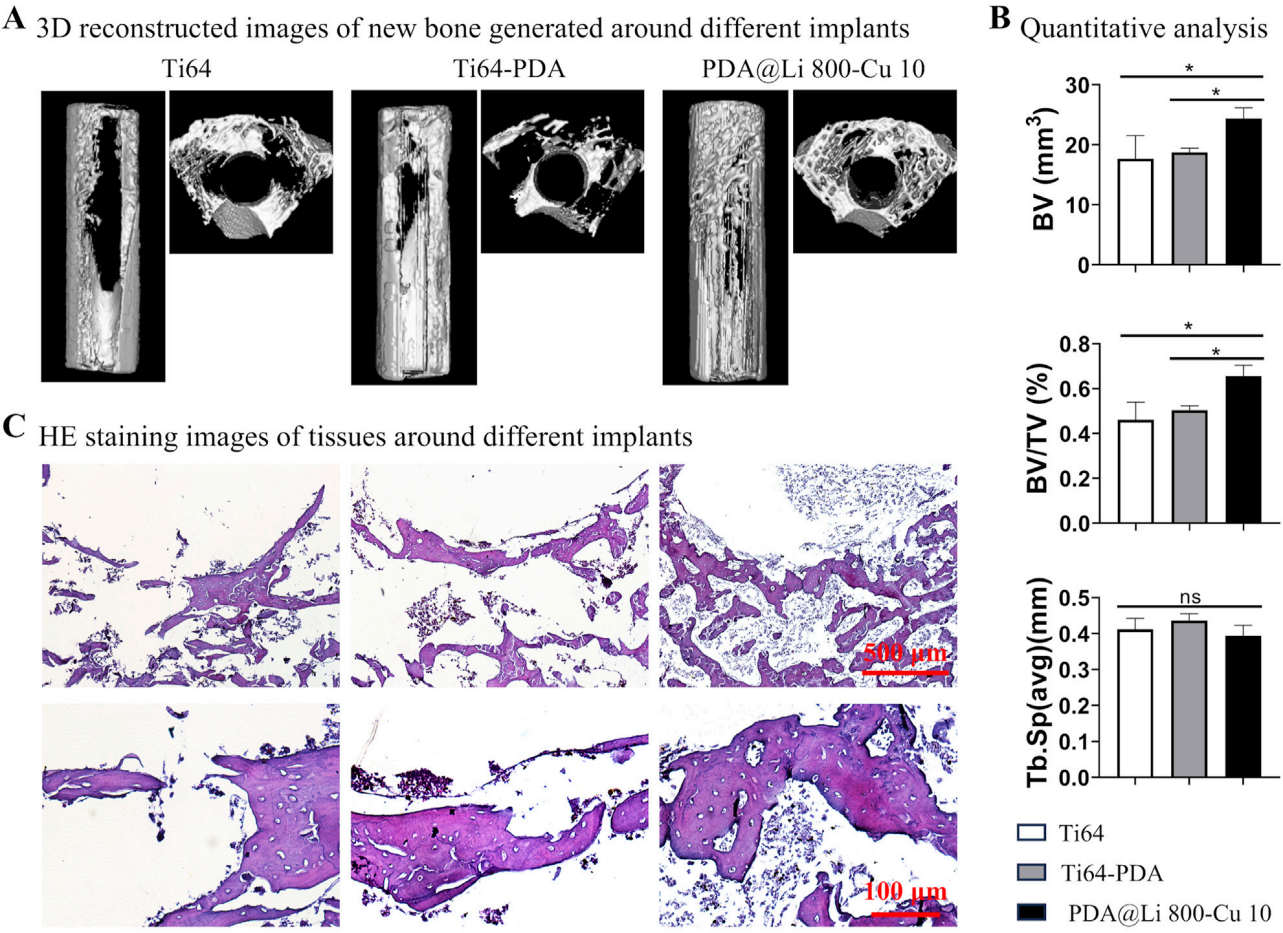
Bone regeneration evaluation after 4 weeks implantation. **(A)** MicroCT reconstructed images of the bone formation around the implanted samples. **(B)** Quantitative results of microCT scanned data. *p < 0. 05 (n = 4). **(C)** HE staining to characterize the osteogenesis associated with various implants. Scale bar: 500 μm (upper), 100 μm (down).

PDA@Li 800-Cu 10 samples with potential anti-infection and osteogenesis capacity were evaluated through micro-CT and histological staining to validate its potential application, polished control and PDA-coated samples were employed as controls (Figure 7). Micro-CT reconstructions illustrate that the new bone formation (indicated in white) at the periphery of the implanted materials has distinctive differences, with more bone observed around the PDA@Li 800-Cu 10 in comparison to controls (Figure 7A). Furthermore, comparative quantitative analysis revealed significantly higher calculated values of bone volume (BV) and bone volume/total volume (BV/TV) for PDA@Li 800-Cu 10, which comforted the observed images (Figure 7B). However, with respect to trabecular separation (Tb.Sp), the values for PDA@Li 800-Cu 10 were slightly lower than other control groups but it is not significant in the statistics. Histological assessment via HE staining further demonstrates the osteogenic activity, showing mature trabecular bone (pink eosinophilic matrix) and minimal fibrotic tissue at the implant-tissue interface of PDA@Li 800-Cu 10. These multimodal analyses collectively highlight the osteoconductive efficacy of the experimental implant.

The dual functionality of copper in bone regeneration–balancing antimicrobial efficacy with osteogenic capacity–is critically influenced by its local concentration, release kinetics, and systemic metabolic thresholds (Burghardt et al., 2015; Xu et al., 2018; Wan et al., 2022; Zhang et al., 2022). The results reported are controversial, Burghardt et al. reported that at low concentrations (≤0.1 mM), Cu^2+^ has a negatable anti-bacterial effect on *S. aureus* but enhanced osteogenesis of MSC evidenced by collagen expression and matrix maturation, this may be attributed to that Cu^2+^ serves as a cofactor for lysyl oxidase, an enzyme essential for collagen crosslinking and bone matrix integrity (Rodríguez et al., 2002). Conversely, elevated concentration (≥0.3 mM) significantly reduced bacterial proliferation, however, the osteogenic differentiation of MSC was also suppressed evidenced by inhibited ALP activity and downregulated osteogenic genes (Burghardt et al., 2015). These biphasic effects mirror systematic copper regulation: serum levels of 98.5–114 μg dL^−1^ optimize bone mineral density (BMD), while deviations correlate with increased fracture risk and reduced cortical strength (Qu et al., 2018), emphasizing the need for precise dosing for Cu^2+^ delivery to avoid uncontrolled Cu^2+^ release irreversibly impairs bone regeneration (Zhang et al., 2022).

Delivery systems critically modulate Cu^2+^ and Li^+^’s bioactivity. Ion-implanted Cu^2+^ in PEEK (Wan et al., 2022), incorporating Cu^2+^ to borosilicate glass bone cement (Li et al., 2023), and alloyed Ti6Al4V-6Cu (Xu et al., 2018) preserved osteoblast function by limiting free ion release, at the same time, demonstrated *in vitro* anti-bacterial capacity and *in vivo* anti-infection efficacy. While polydopamine–medicated codoping with Li^+^ reported in this study rescued *ALP* gene expression and protein activity at the Cu^2+^ levels that have remarkable inhibition on the osteogenic differentiation of MC3T3-E1. This synergy mirrors Li’s capacity to activate Wnt signaling, counteracting Cu^2+^-induced pathway suppression. Clinically, spatial control over Cu^2+^ release–combining high Cu^2+^ peripheries for infection resistance and low Cu^2+^ interfaces for osteointegration–offers a promising strategy. Such designs align with graded coatings and codelivery systems (e.g., Li^+^/Cu^2+^) that optimize both antibacterial and osteogenic timelines. Cu^2+^’s dual functionality demands precision engineering to harness its antibacterial benefits without compromising bone repair. Integrating Cu^2+^ with osteogenic ions (Li^+^, Sr^2+^) and advanced delivery platforms will enable next-generation implants that dynamically adapt to the healing microenvironment.

Recent advances in implant surface engineering have expanded significantly through innovations in (1) fabrication techniques (e.g., plasma spraying, magnetron sputtering, polydopamine (PDA)-mediated coatings) (Cui et al., 2022; Wang Z. et al., 2022; Kheirmand-Parizi et al., 2024; Li B. et al., 2024), (2) bioactive components (metallic ions: Zn^2+^, Cu^2+^, Sr^2+^; non-metallic agents: peptides, antibiotics, metal-organic frameworks (MOFs)) (Pan et al., 2020; Pan et al., 2021; Yu et al., 2023; Kheirmand-Parizi et al., 2024; Lin et al., 2024; Shou et al., 2024; Chen et al., 2025; Zhou et al., 2025), and (3) stimuli-responsive systems (e.g., pH-, reactive oxygen species (ROS)-, inflammation-, and ultrasound-activated coatings) (Chen et al., 2022; Yu et al., 2023; Li S. et al., 2024; Yang et al., 2024; Chen et al., 2025; Yu et al., 2025; Zhou et al., 2025). These advancements aim to achieve multifunctionality, including antibacterial activity, cell recruitment, osteogenic enhancement, and immunomodulatory effects. For instance, Shou et al. demonstrated that RGD/OGP-functionalized metal-polyphenol networks (MPNs) enhance osteoblast differentiation and immune regulation synergistically (Shou et al., 2024). However, despite these innovations, clinical translation remains challenged by competing demands: (1) versatility (adaptability to dynamic physiological microenvironments), (2) manufacturing scalability (avoiding complex vacuum-/energy-intensive processes), and (3) biocompatibility (minimizing cytotoxicity of non-native elements like Ag^+^). Unlike Ag/Zn or Zn/BMP-2 systems, which have uncontrollable cytotoxicity or ectopic bone formation, our PDA-mediated Li/Cu coating leverages endogenous ions to ensure biocompatibility while enabling aqueous, ambient-condition fabrication. This strategy balances efficacy (>95% bacterial suppression), safety (cytocompatibility), and translational practicality, addressing the persistent trade-off between functional complexity and clinical viability inherent to many advanced coatings.

This study demonstrates the successful implementation of a polydopamine-mediated dual delivery system for Cu^2+^ and Li^+^, which synergistically enhances antibacterial efficacy and osteogenic activity. However, several critical limitations warrant further investigation to advance clinical translation. While our findings validate short-term antibacterial performance *in vitro* and infection suppression *in vivo*, the long-term therapeutic particularly against evolving antibiotic-resistant pathogens remains unaddressed. Additionally, despite promising biocompatibility profiles observed in preliminary assessments, rigorous evaluation of systemic biosafety for polydopamine and its combined use with Cu/Li ions is imperative. Current *in vitro* models inadequately replicate the dynamic physiological microenvironment, as evidenced by a European multicenter analysis revealing only 58% covariance between *in vitro* biomaterial testing outcomes and *in vivo* bone regeneration efficacy (Hulsart-Billstrom et al., 2016). This discrepancy likely stems from oversimplified models that neglect host immune responses to foreign implants, a factor poorly represented in conventional evaluations. To bridge this gap, the development of advanced preclinical platforms integrating immunomodulatory assessments (e.g., macrophage polarization, and cytokine profiling) is essential to predict long-term implant performance (Hulsart-Billstrom et al., 2016; Lock et al., 2019). Furthermore, microenvironment-responsive drug delivery systems (e.g., pH-, enzyme-, or light-triggered release) have shown remarkable preclinical success in combating infections and inflammation, the development of such systems to deliver the bioactive ions is one of the future directions. However, their clinical applicability is constrained by the complexity of human pathophysiology (Zhang J. et al., 2024; Chen et al., 2025). For instance, temporal heterogeneity in infection sites and patient-specific metabolic variations challenge the spatiotemporal precision of therapeutic release. Addressing these limitations demands interdisciplinary strategies, including patient-derived organoid models and machine learning-driven pharmacokinetic simulations, to optimize personalized implant designs for translational success (Suwardi et al., 2021; Bai et al., 2024).

## 4 Conclusion

In conclusion, we have engineered a dual-functional titanium alloy (Ti64) implant through PDA-mediated co-incorporation of Li^+^ and Cu^2+^, addressing two critical challenges in orthopedic implants: insufficient osseointegration and implant-associated infections. By optimizing Li (800 mM) and Cu (10 mM) concentrations, the dual-modified surface (PDA@Li 800-Cu 10) synergistically harnesses the osteoinductive properties of Li^+^ and the bactericidal activity of Cu^2+^, while mitigating their limitations. *In vitro*, Li loaded surfaces promoted MC3T3-E1 pre-osteoblast proliferation and osteogenic differentiation as evidenced by upregulated *ALP* and *Axin2* expression, whereas Cu^2+^ confers potent antibacterial efficacy against *S. aureus* and *E. coli*. Crucially, the dual-doped surface balances these functionalities, achieving sustained antibacterial performance without compromising osteogenesis. *In vivo* validation in a rat femoral model demonstrates the translational promise of this strategy: PDA@Li 800-Cu 10 implants eradicated bacterial colonization within 3 days (>80% reduction in *S. aureus*) and drove significant peri-implant bone formation after 4 weeks, with micro-CT revealing a significantly increased bone volume fraction (BV/TV) compared to polished Ti64 controls. This work not only advances a scalable, multifunctional coating strategy but also provides a blueprint for harmonizing biological and antimicrobial functionalities in next-generation implants, with broad implications for orthopedic and dental applications.

## Data Availability

Datasets are available on request: The raw data supporting the conclusions of this article will be made available by the authors, without undue reservation.
