# Supplementary material for: Dual-functional titanium implants via polydopamine-mediated lithium and copper co-incorporation: synergistic enhancement of osseointegration and antibacterial efficacy
Source: Front Bioeng Biotechnol. 2025 May 12;13:1593545. doi: 10.3389/fbioe.2025.1593545 (PMC12104301; doi:10.3389/fbioe.2025.1593545)
Supplement: Supplementary file 1 [file DataSheet1.docx]

**Support Information**

**Dual-Functional Titanium Implants via Polydopamine-Mediated Lithium and Copper Co-Incorporation: Synergistic Enhancement of Osseointegration and Antibacterial Efficacy**

Jun Li*, Bo Jiang, Liu Yang, Pu Zhang, Jingwen Wu, Yalan Yang, Yan Yang, Guiling Wang, Jie Chen, Ling Zhang, Shiqin Huang, Lingli Zhang* and En Zhang*

NMPA Key Laboratory for Quality Monitoring of Narcotic Drugs and Psychotropic Substances, Chongqing Institute for Food and Drug Control, Chongqing, China

*** Correspondence:**
Corresponding Author
[lijun@cqifdc.org.cn](mailto:lijun@cqifdc.org.cn) (J. L.), [zhanglingli@cqifdc.org.cn](mailto:zhanglingli@cqifdc.org.cn) (L. Z), [zhangen@cqifdc.org.cn](mailto:zhangen@cqifdc.org.cn) (E. Z)


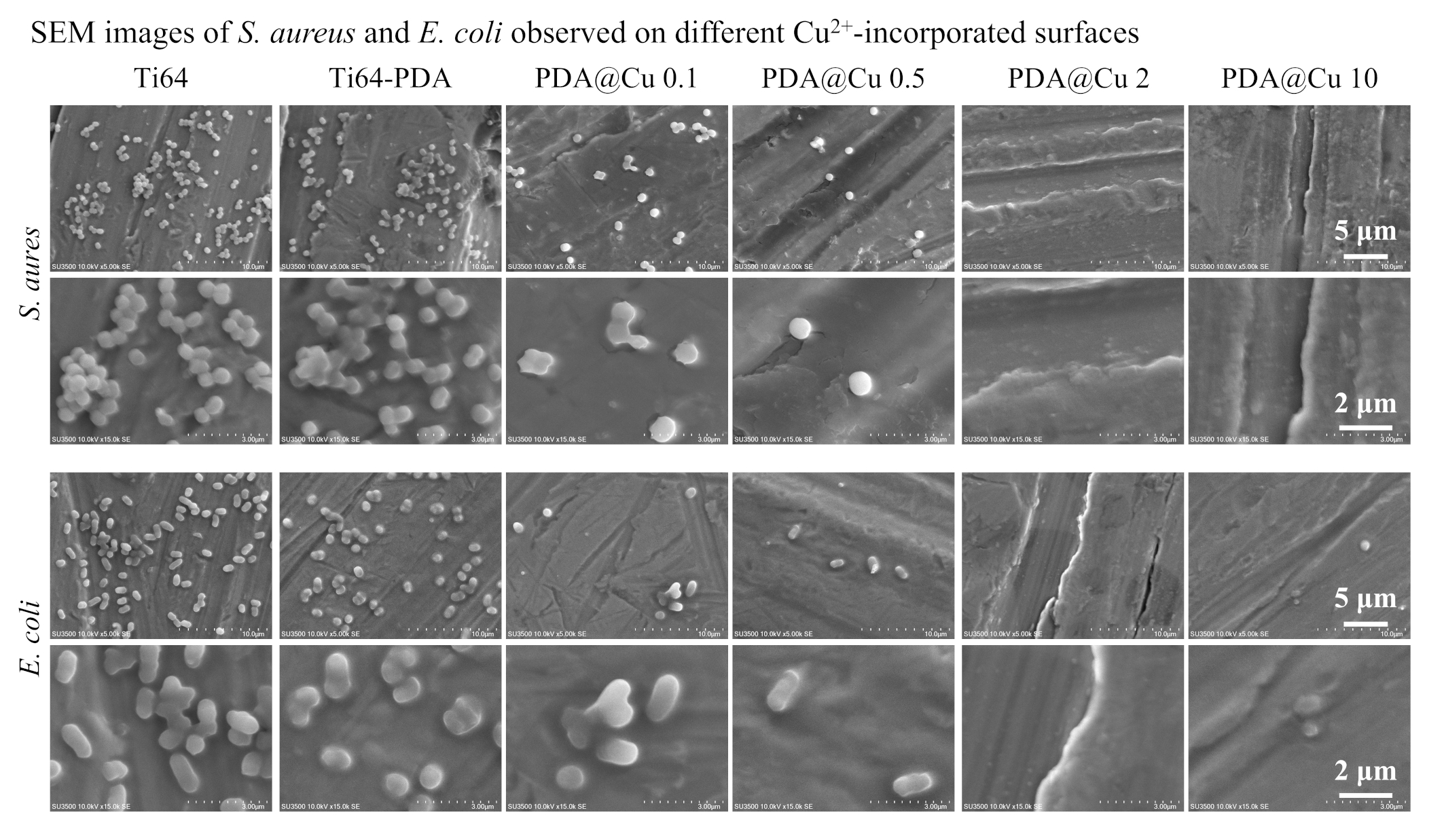
**Figure** **S1.** Representative SEM images of *S. aureus* and *E. coli* after culturing on different Cu^2+^-incorporated surfaces for one day**.**

**
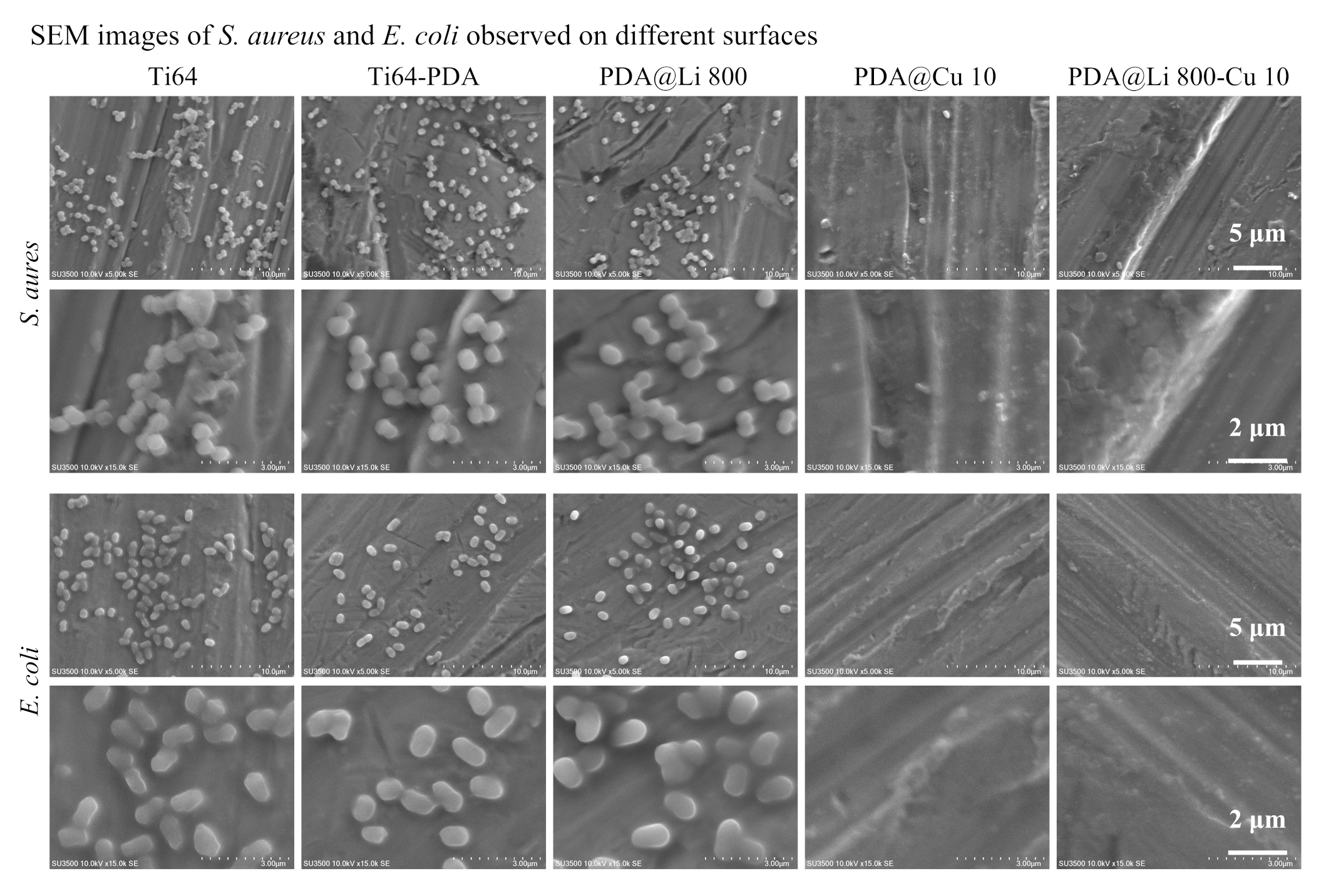
Figure S2.** Representative SEM images of *S. aureus* and *E. coli* after culturing on different surfaces for one day**.**

**
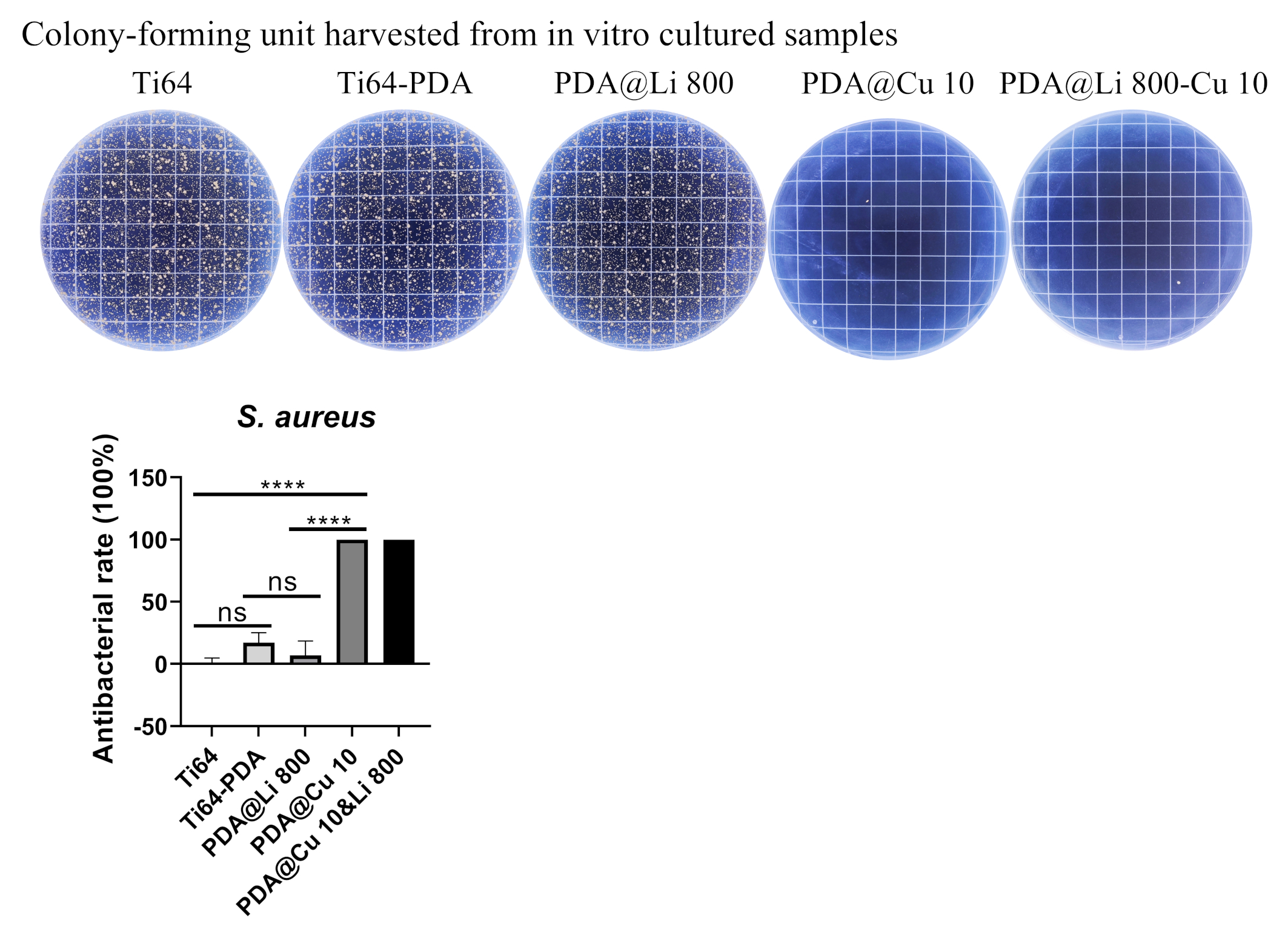
Figure S3.** Representative photographic documentation of *S.aureus* colonies harvested from surfaces that were incubated in PBS for 4 days and corresponding calculated antibacterial rate. *****p* < 0.0001 (n=3).
